# A novel optimized neural network model for cyber attack detection using enhanced whale optimization algorithm

**DOI:** 10.1038/s41598-024-55098-2

**Published:** 2024-03-07

**Authors:** Koganti Krishna Jyothi, Subba Reddy Borra, Koganti Srilakshmi, Praveen Kumar Balachandran, Ganesh Prasad Reddy, Ilhami Colak, C. Dhanamjayulu, Ravikumar Chinthaginjala, Baseem Khan

**Affiliations:** 1 Department of Computer Science and Engineering, Geethanjali College of Engineering and Technology, Hyderabad, TS 501301 India; 2 Department of Information Technology, Malla Reddy Engineering College for Women, Hyderabad, TS India; 3 Department of Electrical and Electronics Engineering, Sreenidhi Institute of Science and Technology, Hyderabad, TS 501301 India; 4 Department of Electrical and Electronics Engineering, Vardhaman College of Engineering, Hyderabad, TS 501218 India; 5 Department of Electrical and Electronics Engineering, AM Reddy Memeorial College of Engineering, Guntur, AP India; 6 Department of Electrical and Electronics Engineering, Faculty of Engineering and Architectures, Nisantasi University, 34398 Istanbul, Turkey; 7 School of Electronics Engineering, Vellore Institute of Technology, Vellore, India; 8 Department of Electrical and Computer Engineering, Hawassa University, Hawassa 05, Ethiopia

**Keywords:** Artificial neural network (ANN), Cyber-attack, Credential stuffing, Energy science and technology, Engineering

## Abstract

Cybersecurity is critical in today’s digitally linked and networked society. There is no way to overestimate the importance of cyber security as technology develops and becomes more pervasive in our daily lives. Cybersecurity is essential to people’s protection. One type of cyberattack known as “credential stuffing” involves using previously acquired usernames and passwords by attackers to access user accounts on several websites without authorization. This is feasible as a lot of people use the same passwords and usernames on several different websites. Maintaining the security of online accounts requires defence against credential-stuffing attacks. The problems of credential stuffing attacks, failure detection, and prediction can be handled by the suggested EWOA-ANN model. Here, a novel optimization approach known as Enhanced Whale Optimization Algorithm (EWOA) is put on to train the neural network. The effectiveness of the suggested attack identification model has been demonstrated, and an empirical comparison will be carried out with respect to specific security analysis.

## Introduction

Because more people are using digital devices that are connected to the Internet, cyber security has grown in importance as a field of study. Although the widespread interconnectedness has made consumers’ lives easier, it has also made them more susceptible to cyber security problems. In order to create countermeasures against the hazards caused by attackers, scientists are concentrating on that field^1^. Organizational networks, information systems, and infrastructures, as well as personal devices and networks, are frequently the targets of hackers. Cyber attacks have advanced significantly since the late 1980s, coinciding with the advancement of technological innovation. Regrettably, this expands the potential “surface” for cyber attacks.

The spectacular factor, the vulnerability factor, and the terror factor are the three factors that drive cyberattacks. The impact or damage that a malevolent attacker can cause is related to the spectacular factor. Damages could include a decline in the target’s visibility as well as a person or organization’s financial loss. For instance, if a Denial of Service assault were to occur, it would cause a loss of revenue since it would disrupt the operations of major e-commerce sites like Amazon, Lazada, or TaoBao. The next consideration has to do with a person or organization’s susceptibility. Some businesses may be operating with antiquated infrastructure and security systems, which make them an easy target for attackers.

A cyber attack known as “credential stuffing” takes advantage of the habit of using the same login and username and password combinations on several different websites. Attackers quickly and methodically enter huge lists of username and password credentials into different websites, apps, and online services using automated technologies, frequently in the form of bots. The intention is to get into user accounts without authorization^2^. Credential stuffing attacks are successful because a lot of people reuse their passwords on several platforms. Since 64% of people reuse their passwords across several accounts—and occasionally all of them—credential stuffing attacks are among the most frequent sources of data breaches. In fact, about half of all login attempts we receive each day on Auth0’s platform alone are attempts at credential stuffing.

**Figure 1:**
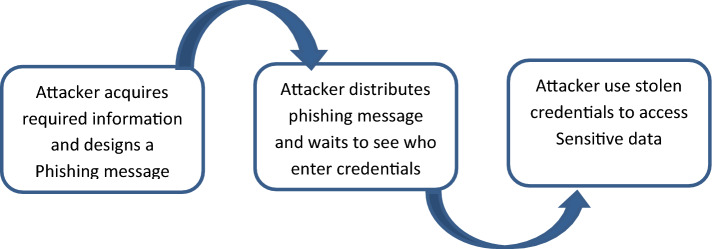
attackers in credential stuffing attacks.

As shown in Fig. 1 If a user’s credentials are compromised in one data breach, attackers may attempt to use the same credentials on other websites or services where the user has an account. To protect against credential stuffing attacks, it’s essential for users to follow good security practices, such as Use Unique Passwords, Enable Two-Factor Authentication (2FA), Regularly Update Passwords, Monitor Accounts etc.

Attackers may try to exploit a user’s credentials on other websites or services where the person has a user account if their credentials are stolen in a breach of data. It’s critical for consumers to adhere to strong security measures, such as using unique passwords, turning on two-factor authentication (2FA), updating passwords frequently, monitoring accounts, etc., in order to defend towards credential stuffing attacks.

The majority of researchers rely on the network’s authentication using cryptography. The authentication process doesn’t reveal whether the attacker is present or not; it only verifies the nodes’ authenticity. Therefore, attack detection that utilizes machine learning is crucial. Several strategies have been used with the Optimization Techniques to overcome the challenges of identifying attack susceptibility.

Attack detection techniques that are commonly utilized these days are intelligent and meta-heuristic approaches. These techniques can be utilized to analyse attack databases and to improve and increase classifier accuracy. As a result, these methods for identifying assaults and abnormalities are trustworthy and suitable. To produce the best outcomes, these methods estimate the multi-objective variable. However, there are a variety of reasons why the optimization method and neural network can be combined. Providing the network with machine learning (MI) has become one of the most essential tasks^3–6^.

The following summarizes the primary contributions of the suggested work:

- Offers a plan that includes an attack identification strategy based on MI with optimization support.
- Describes the concept of optimization for recognizing attack processes that are implemented under the corresponding limitations of energy, penalty, and time.
- Preserves the secrecy component of the suggested attack identification system to ensure reliable and attack free network interaction.
- Offers a novel Enhanced Whale Optimization model, an improved version of the conventional WOA algorithm, for resolving the specified optimization problems.

The rest of the paper isplanned as follows: in Section “Literature review” lists the most significant studies that have been conducted in the relevant literature; Section “Proposed architecture for attacker node identification detection in cyber network using machine learning” describes the structure for the MI-based attack detection system and authentication; Section “Proposed optimization based attack detection system to secure from credential-stuffing attack” talks about the suggested optimization-based attack detection system for secure communication; Section “Results and discussions” shows enhanced neural network model for attack discovery; Section “Conclusion” talks about the outcome of the designed approach; and concludes.

## Literature review

Numerous academics have examined a great deal of work for finding intrusion in this study. Nga et al.^7^ proposal for intrusion detection makes use of several node behavior characteristic features. The authors developed a successful method for detecting network attacks by fusing effective sensor data fusion with precise attack behavior recognition. togather the real-time status information the authors use a lightweight protocols interaction mechanism of both the client and the server, thereby reducing both the frequency of false alarms and the network overhead. Conversely, Mean Daniyar^8^ described the anomalous behavior of the data packet using the FHCA model. The algorithm is a tried-and-true technique for identifying unusual traffic conditions as a disaster develops; attack detection false alarm rate needs to be raised. Thus, a genetic algorithm is introduced by Hoque et al.^9^ for the intrusion detection system. Using the KDD99 benchmark dataset, the authors used the idea of information to filter traffic data and simplify the process. In order to combine four distinct detection techniques, Mangrulkar et al.^10^ employed DDoS assaults. There is no reliable application layer detection mechanism in place; this approach is solely utilized for network layer protocols. A security framework against DoSattacks in peer-to-peer systems was devised by Cusack and Almutairi^11^. On the other hand, Zho et al.^12^ put forth the theory to ascertain how natural text samples behave. This system aims to monitor irregular behaviour of the network that departs from standard grammatical rules, is established using an enhanced hidden Markov model. In conclusion, Chen et al.^13^ presents an enhanced WOA (WOAmM) is proposed. The mutualism phase from Symbiotic Organisms Search (SOS) is modified and integrated with WOA to alleviate premature convergence’s inherent drawback. The addition of a modified mutualism phase with WOA makes the algorithm a balanced one to explore search space more extensively and avoid wasting computational resources in excessive exploitation. Abiodun et al.^14^ and Omolara et al.^15^ presents a survey on Cyber-attacks have evolved into a type of asymmetrical warfare that is of great concern not only to computer scientists but also to the international community. Abiodun et al.^16^ Proposed a feed forward and feedback propagation ANN models for research focus based on data analysis factors like accuracy, processing speed, latency, fault tolerance, volume, scalability, convergence, and performance. Alawida et al.^17^ Presents a survey that shows differences in cyber-attack techniques; as hacking attacks was the most frequent with a record of 330 out of 895 attacks, accounting for 37%. Next was Spam emails attack with 13%; emails with 13%; followed by malicious domains with 9%. Mobile apps followed with 8%, Phishing was 7%, Malware 7%, Browsing apps with 6%, DDoS has 6%, Website apps with 6%, and MSMM with 6%. BEC frequency was 4%, Ransomware with 2%, Botnet scored 2% and APT recorded 1%. Taofeek et al.^1^ presents a Cognitive Deception Model (CDM) based on a neural model which takes an input message and generates syntactically cohesive and semantically coherent independent looking but plausible and convincing decoy messages to cognitively burden and deceive the adversaries. The experimental results used to validate the models, as well as the comparison with state-of-the-art tools, show that it outperforms existing systems. Giluka et al.^18^ present intrusion detection for traffic on the network called “Correlation-based Feature-Selection-Bat-Algorithm” (CFSBA). To train and test this algorithm utilizes features of KDDCup99 dataset. This research, offer the whale optimization detection method (DMWO), to compute the standard deviation during the distribution procedure in order to assess the abnormality of the data packet. The primary components of the DMGO simulation algorithm are carried out using OPNET and Matlab-2015a to categorize the input and determine if an attack is there or not.

## Proposed architecture for attacker node identification detection in cyber network using machine learning

**Figure 2:**
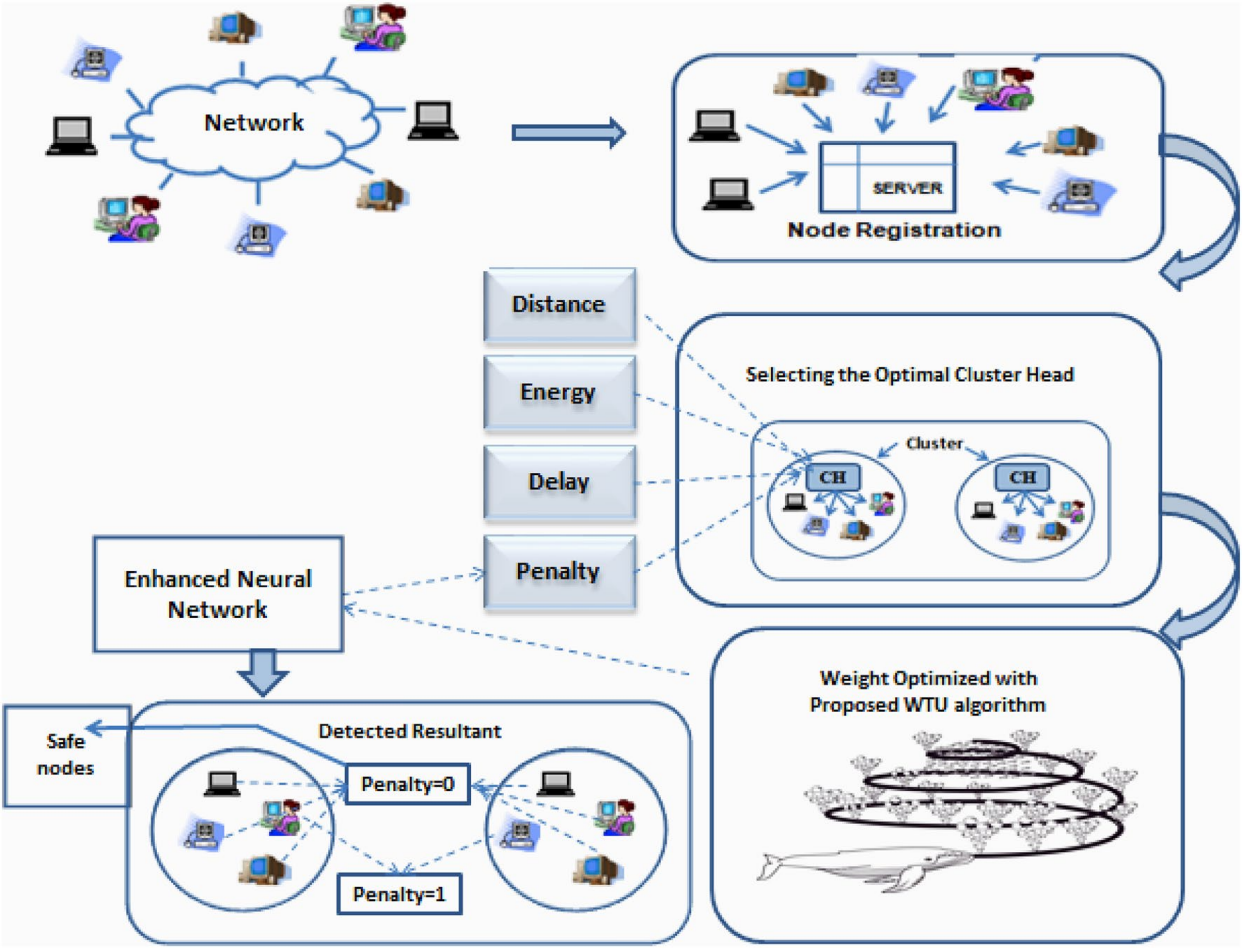
Proposed attack detection framework.

In the proposed framework for the attack detection method in the network is shown in Fig. 2. First, 100 attacker nodes and 100 safe nodes—are included in the KDD Cup dataset and all these nodes are enrolled in the server using unique biometric data. Using a novel Enhanced Whale Optimization model, the CHs are chosen from these nodes. In addition, four criteria are taken into account when making the decision: “distance, penalty, energy, and delay”. The node that has the most energy, lowest distance, penalty, and delay has the chance to function as a CH. As a matter of fact, the clusters are produced based on CH in terms of low proximity to CH and energy below the CH threshold. Additionally, the block chain stores the node data and the ideal CH. After nodes enrolled in to the -network, The cluster are formed to reduce the burden of the network, if an attacker node is found in the process it is difficult to find a node among all these nodes. Instead, if the clusters are created and the cluster head are elected the computation time for finding the node get reduced as the attack node information is shared to the cluster heads. both the CH and Nodes communication continue and the subsequent Neural network attack detection gets performed The penalty function is added with a value of 1, if the attacker is found during the detection phase, otherwise it is zero. The nodes enter the communication process together with CH that has no consequences. The suggested EWOA is unusual in that it chooses the best weights to train the NN model.

## Proposed optimization based attack detection system to secure from credential-stuffing attack

### Optimal CH selection

In this paper, limitations such as “distance, penalty (security), energy, and delay” were taken into account while choosing the CH. As stated, the goal is to identify a CH node, which consists of the lowest distance, penalty, delay, and with higher energy.

*Distance Measurement (*$D$*):* when the nodes come closest to cluster head then clusters are formed. As a result, clusters emerge. The distancematrix $D(m*n)$ is expressed arithmetically using Eq. (1).

1 $$D(m*n) = \left[ \begin{gathered} d_{{M_{c1} ,x_{1} }} d_{{M_{c1} ,x_{2} }} \ldots \ldots d_{{M_{c1} ,x_{n} }} \hfill \\ d_{{M_{c2} ,x_{1} }} d_{{M_{c2} ,x_{2} }} \ldots \ldots d_{{M_{c2} ,x_{n} }} . \hfill \\ : \hfill \\ : \hfill \\ d_{{M_{cm} ,x_{1} }} d_{{M_{cm} ,x_{2} }} \ldots \ldots d_{{M_{cm} ,x_{n} }} \hfill \\ \end{gathered} \right]$$

$d_{{M_{c} }}$ denotes the Euclidean distance among the CH $\left( {M_{c} } \right)$ and the node in Eq. (1). In an LTE network, the sensor nodes are designated as $Y_{1} ,Y_{2} , \ldots Y_{n}$. Furthermore, two nodes’ positions are denoted by $y$ and $z$, and the Euclidean distance $\left( {d_{r,q} } \right)$ is calculated using Eq. (2).

2 $$d_{r,q} = \sqrt {(r_{y} - q_{y} )^{2} + (r_{z} - q_{z} )^{2} }$$

Each element in Eq. (1) indicates the distance far among the node and the $r{\text{th}}$ CH. The distance that a $q{\text{th}}$ node can be linked to a cluster within is known as the threshold distance in numbers. the packet transmission $F_{(a)}^{D}$ from the cluster head to the node and then from the CH to the base station.

The $Y_{y}$ normal node that is a part of the cluster and the CH of the $y{\text{th}}$ cluster are represented by the notation $c_{y}$. Additionally, the far among CH and the normal node is given by $c_{y} - A_{s}$, and the distance between BS and CH is represented by $c_{y} - Y_{y}$. $Y_{y} - Y_{z}$ the separation among two normal nodes in Eq. (5). In this case, the total node count is associated with the clusters $z{\text{th}}$ and $y{\text{th}}$, which are represented by $M_{y}$ and $M_{x}$, respectively. Equations (3), (4), and (5) show the fitness function for distance $\left( {F_{i}^{D} } \right)$.

3 $$F_{i}^{D} = \frac{{F_{(a)}^{D} }}{{F_{(b)}^{D} }}$$

4 $$F_{(a)}^{D} = \sum\limits_{y = 1}^{{M_{y} }} {\left[ {\left\| {c_{y} - A_{s} } \right\| + \sum\limits_{y = 1}^{{M_{z} }} {\left\| {c_{y} - Y_{y} } \right\|} } \right]}$$

5 $$F_{(b)}^{D} = \sum\limits_{y = 1}^{{M_{y} }} {\sum\limits_{z = 1}^{{M_{z} }} {\left\| {Y_{y} - Y_{z} } \right\|} }$$

*Energy model (*$En$*):* One crucial factor to select CH is energy utilization. “The model of the network that reduces energy in various operations such as transmission, reception, sensing, and aggregation is declared by the energy consumption model”. Eq. (6) provides the numerical value of the total energy $\left( {En_{TX} (M:d)} \right)$ required to transport N-bit of data at $d{\text{th}}$ a distance from nodes to cluster head and vice versa. This cutoff distance is shown in Eq. (7). Equation (8) specifies the energy consumed by the node to receive data from the CH and vice versa.

6 $$En_{TX} (M:d) = \left\{ \begin{gathered} En_{el} *M + En_{fs} *M*d^{2} ,if\,\,d < d_{0} \hfill \\ En_{el} *M + En_{pw} *M*d^{2} ,if\,\,d \ge d_{0} \hfill \\ \end{gathered} \right.$$

7 $$d_{0} = \sqrt {\frac{{En_{Fs} }}{{En_{pw} }}}$$

8 $$En_{RX} (M:d) = En_{e} M$$

Furthermore, Eq. (9) displays the energy utilized in the amplification $\left( {En_{am} } \right)$ procedure, and Eq. (10) displays the network’s overall energy cost. The energy cost in the sensing and idle modes is represented by $En_{1}$ and $En_{S}$ r, respectively.

9 $$En_{am} = En_{fs} d^{2}$$

10 $$En_{total} = En_{TX} + En_{RX} + En_{1} + En_{S}$$

Equation (11), where represents arrogation data energy, provides the arithmetical expression for electronic energy $\left( {En_{el} } \right)$. “In which $En_{ae}$ the entire cluster head cumulative $F_{(a)}^{En}$ and $F_{(b)}^{En}$ assumes energy to be of maximum value and the cluster head’s highest count, therefore the value of becomes $F_{i}^{En}$ bigger than one”, according to Eq. (12), is the energy fitness function.

11 $$En_{el} = En_{TX} + En_{ae}$$

12 $$F_{i}^{En} = \frac{{F_{(a)}^{En} }}{{F_{(b)}^{En} }}$$

Delay function ($L$): To determines the fitness function for delay depends on the count of nodes in a cluster. As a result, nodes that exhibit excessive latency are eliminated from groups. The delay’s numerical formula can be found in Eq. (13). The total number of nodes is indicated by $N_{c}$.

13 $$F_{i}^{L} = \frac{{\max \left( {\left\| {c_{x} - Y_{x} } \right\|} \right)_{y = 1}^{{M_{c} }} }}{{M_{c} }}$$

The most restriction of $F_{i}^{L}$ for CH should lie down within 0 to 1.

*Penalty function* $\left( P \right)$ One crucial factor that determines whether a node is an attacker or not is the penalty function. A “1” or a “0” is assigned to the punishment. The Neural Network designates the penalty function as “1” if the nodeis found to be an attacker, and as a result, it is excluded from the MTC process. The penalty function fitness is indicated by the symbol as and the outcome from NN is represented as $F_{i}^{P}$.

The penalty function, determined by a Neural Network, plays a crucial role in identifying attackers among nodes. The binary nature of the penalty function (1 or 0) influences whether a node is excluded from the MTC process. The fitness of the penalty function, indicates its effectiveness, and the outcome from the Neural Network is integral to this determination.

### Optimized neural network

The framework of Neural Network^19^ is used in this work to identify node attacks. The values of 1 or 0 will be allocated to the penalty function based on the result obtained from NN. NN receives the CH $\left( {c_{i} = c_{1} ,c_{2} , \ldots ,c_{n} } \right)$ and the nodes’ $\left( {Y_{i} = Y_{1} ,Y_{2} , \ldots ,Y_{n} } \right)$ behaviour as input for the purpose of detecting attacks. Together, the node performance and CH are represented as. In general, "input, output, and hidden layers" are included in NN. The neurons in the output layer and those in the layer are denoted by $i$ and $j$. The results of hidden layer’s are denoted by $e^{\left( H \right)}$, and they are calculated using Eq. (14).

14 $$e^{\left( H \right)} = nf\left( {W_{{\left( {Bi} \right)}}^{\left( H \right)} + \sum\limits_{j = 1}^{{n_{i} }} {W_{{\left( {ji} \right)}}^{\left( H \right)} f(Y,c)} } \right)$$

The $nf$ activation function and the hidden neuron’s and $W_{{\left( {Bi} \right)}}^{\left( H \right)}$ bias weight are specified in Eq. (28). The number of input neurons $n_{i}$ and the weight of each input neuron $\left( {j{\text{th}}} \right)$ relative to the hidden neuron $\left( {i{\text{th}}} \right)$ is $W_{{\left( {ji} \right)}}^{\left( H \right)}$. Equation (15) is used to evaluate the NN output.

15 $$Q_{o} = nf\left( {W_{{\left( {Bo} \right)}}^{\left( G \right)} + \sum\limits_{i = 1}^{{n_{h} }} {W_{{\left( {io} \right)}}^{\left( G \right)} e_{{}}^{\left( H \right)} } } \right)$$

In this case, the hidden neuron count is $n_{h}$ and the output neurons are denoted as $o$. $W_{{\left( {Bo} \right)}}^{\left( Q \right)}$ signifies the output neurons’ bias weight in addition. Moreover, from the $i{\text{th}}$ hidden neuron to the $o{\text{th}}$ output neuron, the hidden neuron weight is given by $W_{{\left( {io} \right)}}^{\left( Q \right)}$. A penalty function of one (1) is associated with the concerned node or CH if it is found that the node or CH is an attacker; otherwise, the penalty is zero. The evaluation of the discrepancy between the actual and anticipated values is based on Eq. (16).

16 $$E^{ * } = \mathop {\arg \min }\limits_{{\left\{ {W_{{\left( {Bi} \right)}}^{\left( H \right)} ,W_{{\left( {ji} \right)}}^{\left( H \right)} ,W_{{\left( {Bo} \right)}}^{\left( Q \right)} ,W_{{\left( {io} \right)}}^{\left( Q \right)} } \right\}}} \sum\limits_{ = 1}^{{n_{Q} }} {\left| {Q_{o} - \hat{Q}_{o} } \right|}$$

The output neuron count is indicated $n_{Q}$ in Eq. (30), $Q_{o}$ and $\hat{Q}_{o}$ together with the actual and expected output in that order. As previously stated, the suggested EWOA algorithm optimizes the weights = $W$ = $W_{{\left( {Bi} \right)}}^{\left( H \right)}$, $W_{{\left( {ji} \right)}}^{\left( H \right)}$, $W_{{\left( {Bo} \right)}}^{\left( G \right)}$ and $W_{{\left( {io} \right)}}^{\left( G \right)}$ and to train the NN model.

### Solution encoding and objective function

The specific goal of the current study is to select CH by lowering the aim in Eq. (17), where value should fall among $0 < \beta < 1$; $F_{b}$ values are then assessed in accordance with Eqs. (18) and (19), respectively.

17 $$f_{n} = \beta F_{b} + (1 - \beta )F_{a}$$

18 $$F_{a} = \sigma_{1} *F_{i}^{D} + \sigma_{2} *F_{i}^{En} + \sigma_{3} *F_{i}^{L} + \sigma_{4} *F_{i}^{P}$$

19 $$F_{b} = \frac{1}{n}\sum\limits_{x = 1}^{n} {\left\| {M_{Y} - A_{s} } \right\|}$$

The fixed parameters denoted by $\sigma_{1}$, $\sigma_{2}$, $\sigma_{3}$ and $\sigma_{4}$, in those order, are energy ($En$), distance ($D$), delay ($L$), and penalty ($P$). The state must be followed through these constant parameters.

### Proposed enhanced whale optimization algorithm

Modifications to the algorithm are proposed in order to increase the convergence rate and speed performance of the current WOA^20^. The Whale Optimization Algorithm is designed for fast convergence to near-optimal solutions. This quick convergence is valuable in applications where computational resources are limited, or where timely decision-making is critical. It has been demonstrated that self-improvement can be effective in conventional optimization techniques^21–25^. This is a brief explanation of the suggested EWOA algorithm’s mathematical modeling.

i. Prey Encircling: The whales are able to locate their prey and circle around them. Equations (20) and (21), the coefficient vectors are $\vec{B}$ and $\vec{H}$ and ongoing iteration is represented with $t$, provide the surrounding actions of humpback whales.

20 $$\vec{G} = \left| {\vec{H}.\vec{R}_{p} \left( t \right) - \vec{R}\left( t \right)} \right|$$

21 $$\vec{R}\left( {t + 1} \right) = \vec{R}_{p} \left( t \right) - \vec{B}.\vec{G}$$

Furthermore, $\vec{R}$ denotes the position vector and $\vec{R}_{p}$ denotes the best position that has been found thus far. Additionally, $\vec{B}$ and $\vec{H}$ are determined using Eqs. (22) and (23). The component in Eq. (24) decreases for different iterations from 2 to 0. The random vectors $ra_{1}$ and $ra_{2}$ locations are in the interval [0, 1].

22 $$\vec{B} = 2\overset{\lower0.5em\hbox{$\smash{\scriptscriptstyle\frown}$}}{a} .ra_{1} - \vec{a}$$

23 $$\vec{H} = 2ra_{2}$$

(i) Exploitation phase

The “Shrinking encircling mechanism and Spiral updating position” are the foundation for this phase’s modelling.

- *“Encircling Shrinking system”: *This was achieved by reducing the value in Eq. (24).
- *New Spiral update Evaluation with Tri-level:*

Within the position of the $i{\text{th}}$ whale and the prey by Eq. (26) a spiral formula is formed, $\vec{G}$ which denotes the distance that occurs between them and $l$ is an integer that falls among and is a $b$ parameter that sets the logarithmic spiral shape. Equation (25) gives the mathematical expression for.

24 $$R(t + 1) = \vec{G}^{\prime } \,e^{bl} .{\text{Cos}} (2\pi l) + \vec{R}_{p} (t)$$

25 $$G\vec{^{\prime}} = \left| {\vec{R}_{p} (t) - \vec{R}(t)} \right|$$

Whale positions are quantitatively displayed during optimization in Eq. (26),

26 $$R(t + 1)\, = \left\{ {\begin{array}{*{20}l} {\vec{R}_{p} (t) - \vec{B} \cdot \vec{G}\,\,\,\,\,\,\,if\,} \hfill & {\phi < {0}{\text{.5 }}} \hfill \\ {G\vec{^{\prime}}{\prime} \cdot e^{bl} \cdot {\text{Cos}} (2\pi l) + \vec{R}_{p} (t)\,\,\,\,if\,} \hfill & {\phi \ge 0.5} \hfill \\ \end{array} } \right.$$

This version integrates an innovative tri-level update in addition to the standard update evaluation. Initially, the values of $\phi_{1}$, $\phi_{2}$ and $\phi_{3}$, and are set. If, $\phi < 0.5$ then Eqs. (26) and (21) are used to calculate the values $\phi_{1}$ and $\phi_{2}$. Alternatively, $\phi_{3}$ is computed using Eq. (24). Next, a random variable’s $ran$ value is initialized, and if, $ran\; \le \;0.3$ the search agent position is updated using Eq. (25). If $ran = 0.3\,to\,0.6$ the current search agent position is modified in accordance with Eq. (26). In the event that these two requirements are not met, the current search agent position is modified in accordance with Eq. (27–29). Since there are three stages of updating, the chosen technique is known as EWOA.

27 $$\vec{R}(t + 1) = \frac{{\phi_{1} + \phi_{2} }}{2}$$

28 $$\vec{R}(t + 1) = \frac{{\phi_{2} + \phi_{3} }}{2}$$

29 $$\vec{R}(t + 1) = \frac{{\phi_{1} + \phi_{3} }}{2}$$

(ii) Search for Prey (Exploration Phase): The evaluation of this is provided by Eqs. (30) and (31). The vector representing the arbitrary position chosen from the current population is represented by $X_{(rand)}$.
  30 $$\vec{G} = \left| {\vec{H}\,\vec{R}_{(rand)} - \vec{R}} \right|$$
  31 $$\vec{R}(t + 1) = \vec{X}_{(rand)} - \vec{B} \cdot \vec{G}$$

## Results and discussions

The suggested technique for detecting attacks in cyber networks by utilizing an optimization method was evaluated in MATLAB, and the outputs obtained are documented. In terms of alive nodes and network lifetime, the suggested model is contrasted with more established models such as firefly algorithm FF^25^, Jaya algorithm JA^24^, grey wolf with jaya algorithm WI-JA approach^26^ and Grey wolf algorithm GWO^19^, The work that has been provided is useful for assessing live nodes and extending network life.

### Analysis on alive nodes

**Figure 3:**
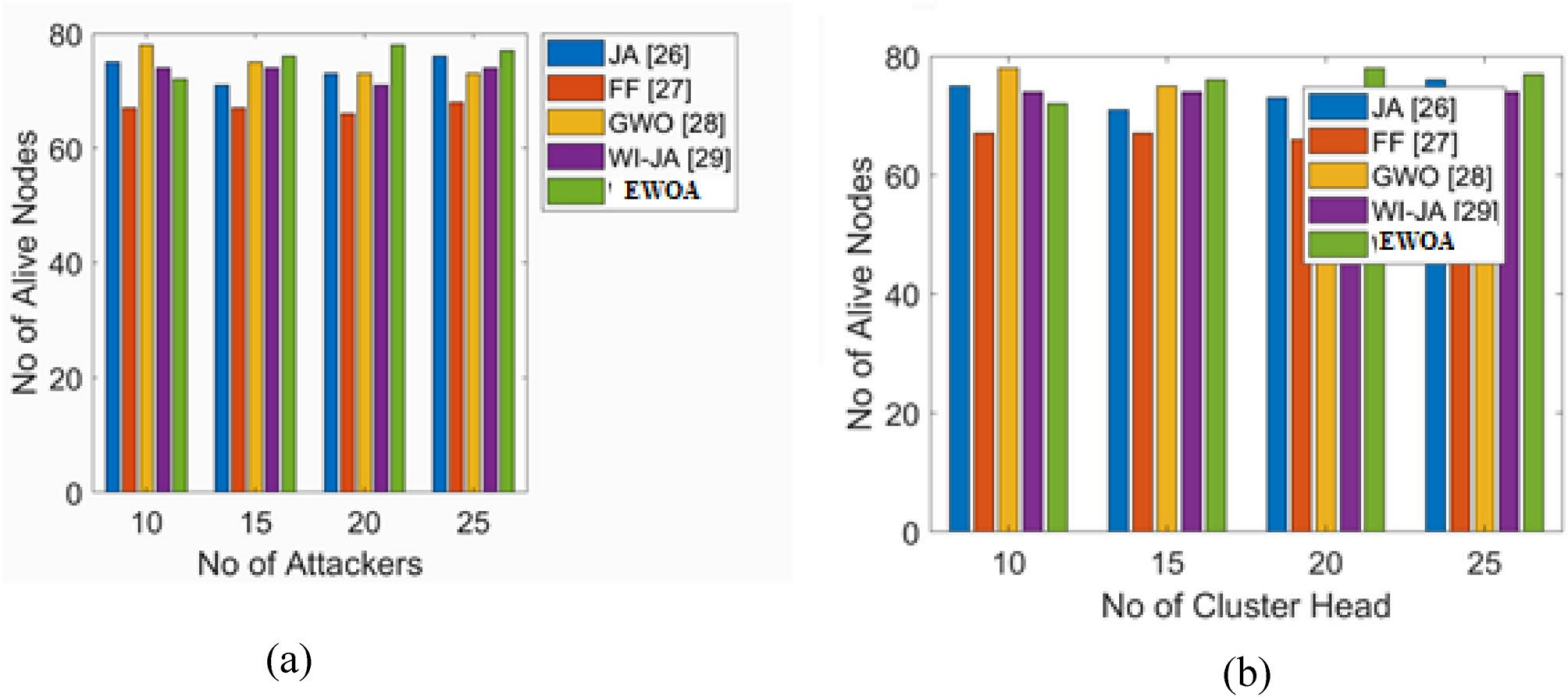
Alive nodes analysis. (**a**) count of attackers and (**b**) count of CH.

The safe nodes that remain at the conclusion every round are known as the alive nodes. Here, a 100-round evaluation of the work that has been put forward as well as the works that already exist is conducted, and the end results are graphically displayed in Fig. 2. By changing the attacker and CH counts, the number of live nodes at the end of each round is calculated. By altering the number of attackers in the network, Fig. 3a projects the number of the alive nodes at the last of the 100th iteration. The suggested work continues to have more live nodes when the attacker count reaches 20. better than the conventional models like FF, JA, WI-JA, and GWO in that order.

The examination makes clear that, the count of live nodes produced by the task that is being given is higher even in the presence of more attackers. Furthermore, the count of alive nodes obtained for the various counts of CHs is in Fig. 3b. The number of CH tends to fluctuate since the nodes eventually run out of energy and die, and because more nodes are constantly joining the network. Since 100 nodes are selected for this work, the CH count must be 10 or above. The count of alive nodes in the work that is being presented is high when the count of CH = 15; when compared to the other techniques in that order. As a result, it is evident from the evaluation that, for the given job with the variable number of CHs, alive nodes number is more.

### Evaluation on network lifetime

**Figure 4:**
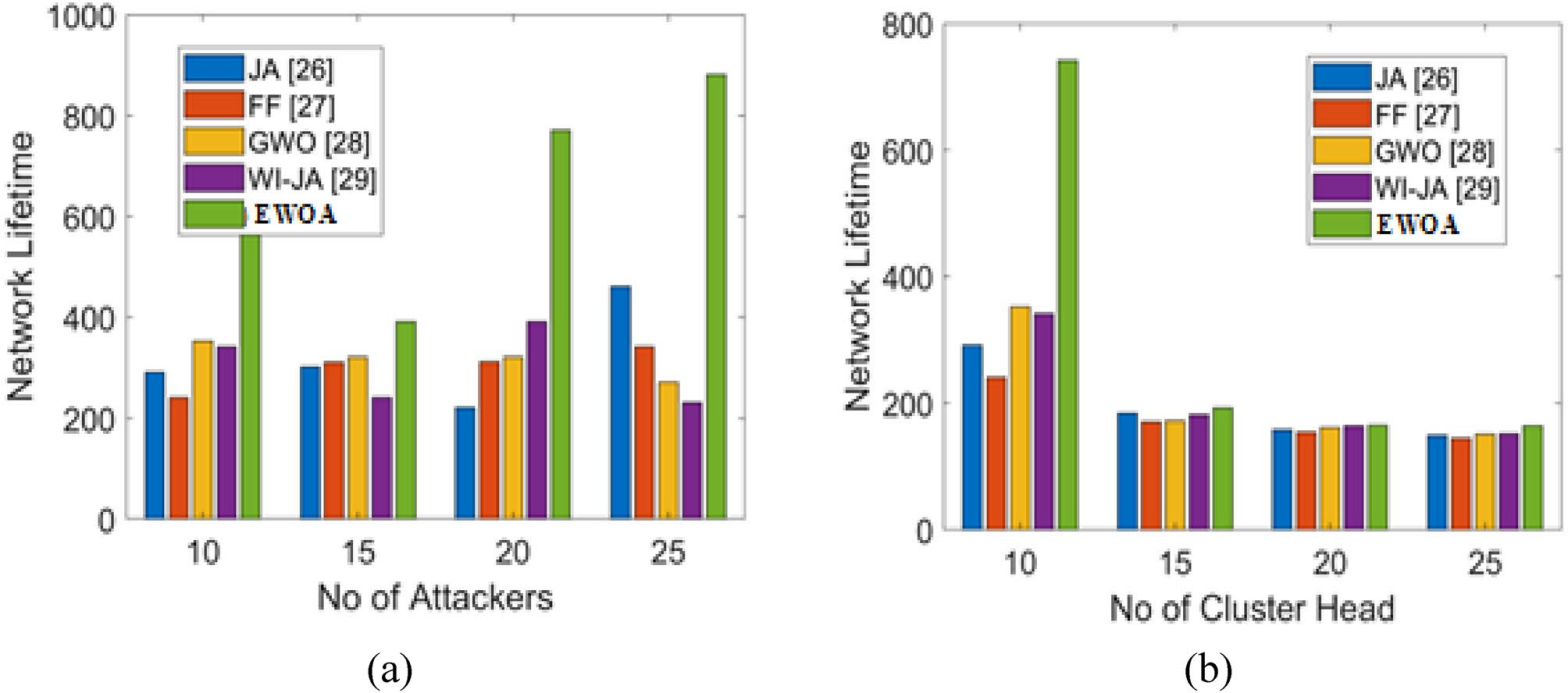
Analysis on network lifetime. (**a**) Attackers count, (**b**) CH count.

The lifetime ration is computed in order to keep a network stable and provide the necessary capacity for sending data packets within the network. One crucial factor in determining performance is the lifetime ratio. The performance assessment of the work being done over the traditional procedure is shown in Fig. 4, where the attackers in count and CHs is varied. The outcomes of network life for the various attacker node counts are shown in Fig. 3a. When compared to other standard models, it is found that the network lifetime utilizing the suggested approach increases even in the presence of more attacker nodes.

More specifically, the suggested model achieves a high network lifetime when the attacker count is equal to 25, Furthermore, Fig. 4b illustrates the network lifespan analysis for a range of CH counts utilizing the work that has been presented. Compared to the standard models, the provided work has the greatest network lifetime, according to the evaluation’s overall findings.

### Statistical performance evaluation

Since the meta-heuristic algorithm is stochastic in nature, and to ensure a fair comparison, each algorithm is executed ten times to obtain the statistics of the number of alive nodes, normalized network energy and the objective cost to be minimized. This evaluation is undergone for a varying count of CH’s and the resultants are tabulated in Table 1. The analysis is carried out under different cases like best, worst, mean, median and standard deviation. In the case of the best-case scenario, the presented work is 3.2%, 1.4%, 5.3%, and 3.1% better than the traditional models like JA, FF, GWO, and WI-JA, respectively.

**Table 1:** Statistical evaluation of presented work over the existing works: accuracy.

Count of CH	JA^24^	FF^25^	GWO^19^	WI-JA^26^	EWOA
Best performance					
CH = 10	0.002998	0.002893	0.002862	0.002769	0.002764
CH = 15	0.004151	0.004522	0.004174	0.004249	0.004207
CH = 20	0.005673	0.005438	0.005924	0.00577	0.005095
CH = 25	0.006933	0.007058	0.006798	0.006942	0.007059
Worst performance					
CH = 10	0.003731	0.003725	0.003702	0.003675	0.003523
CH = 15	0.00553	0.005687	0.005757	0.005347	0.005258
CH = 20	0.007312	0.00753	0.007323	0.007267	0.007235
CH = 25	0.009237	0.009535	0.009186	0.009124	0.008936
Mean					
CH = 10	0.00337	0.003359	0.003308	0.003299	0.003182
CH = 15	0.004992	0.005144	0.005037	0.004939	0.004777
CH = 20	0.006674	0.006862	0.006613	0.006561	0.006412
CH = 25	0.008309	0.008538	0.008335	0.008249	0.008223
Median					
CH = 10	0.003377	0.003363	0.003325	0.003304	0.003189
CH = 15	0.005033	0.005166	0.005039	0.004951	0.004821
CH = 20	0.006715	0.006906	0.006653	0.006547	0.006401
CH = 25	0.008369	0.008607	0.008364	0.008357	0.008245
Standard deviation					
CH = 10	0.000156	0.000195	0.000172	0.000178	0.000164
CH = 15	0.000253	0.000259	0.000311	0.000228	0.000148
CH = 20	0.000366	0.000369	0.000323	0.000318	0.000248
CH = 25	0.00046	0.000481	0.000487	0.000433	0.000317

In addition, the mean of the presented work is 2.3%, 5.1%, 3.1%, and 1.2% better than the existing works like JA, FF, GWO, and WI-JA, respectively. Thus, from the valuation, it is clear that the accuracy of attack detection in NN is higher.

### Computational analysis

Table 2 displays the computational analysis of the presented work compared to the traditional efforts. When compared to traditional approaches like Jaya algorithm (JA), firefly algorithm (FF), grey wolf with jaya algorithm (WIJA), and grey wolf algorithm (GWO)the proposed model has a shorter computing time, according to the overall analysis. However, cyber security is a dynamic field where threats and vulnerabilities constantly evolve. WOA, being a static optimization algorithm, may not adapt well to dynamic changes in the cyber landscape. New attack strategies or changes in the system’s configuration may pose challenges for WOA.

**Table 2:** Computational analysis comparison.

Methods	Computational time
JA	2395.1
FF	5957.7
WIJA	2731.6
GWO	2691.3
FF	5957.7
WIJA	2731.6
EWOA	2031.8

## Conclusion

In this article, an attack detection model based on MI with optimization support was combined with a cluster-based authentication mechanism. The processes of attack detection and clustering both used the idea of optimization. Four main criteria were taken into consideration when choosing CH: distance, energy, penalty, and delay. The suggested assault detection mechanism for reliable and unaffected network communication. The EWOA, a novel EWOA method, was developed to address the specified optimization problems. The effectiveness of the attack detection model that was presented was demonstrated, and the comparison was completed with respect to specific security analysis. More specifically, the suggested model achieves a high network lifetime when the attacker count is equal to 25, which is 54%, 59%, 64%, and 69% better than the current models, The number of alive nodes in the work that is being presented is high when the count of CH = 15; it is 12.4%, 18.74%, 6.24%, and 4% higher than other. The problems of credential stuffing attacks, failure detection, and prediction is been handled by the suggested EWOA-ANN model successfully.

## Data Availability

The data used to support the findings of this study are included in the article.
